# Advances in Population-Based Healthcare Research: From Measures to Evidence

**DOI:** 10.3390/ijerph192013122

**Published:** 2022-10-12

**Authors:** Pietro Ferrara, Luciana Albano

**Affiliations:** 1 Center for Public Health Research, University of Milan–Bicocca, 20900 Monza, Italy; 2 IRCCS Istituto Auxologico Italiano, 20145 Milano, Italy; 3 Department of Experimental Medicine, University of Campania “Luigi Vanvitelli”, 80138 Naples, Italy

## 1. Foreword

Whether “population health” encompasses a concept of health or a field of study of health determinants is not yet defined, though the term is widely used in healthcare and research worldwide [1]. Commonly, it emphasizes health outcomes’ distribution in specific populations and groups [1,2]. From a broader point of view, this definition focuses on measurement, highlighting the importance of measures of health outcomes as actionable metrics that lead to community health improvements [2].

By focusing more on groups rather than on individual patients, population health is crucial for promoting improvements in the quality of health services through evidence-based decisions, the correction of social and economic determinants of health, and health expenditure revisions.

**Figure 1:**
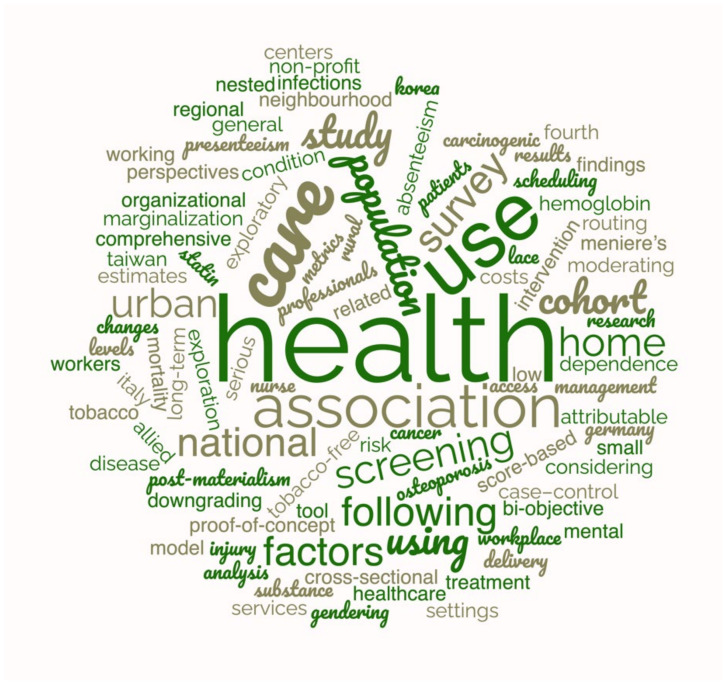
Word cloud based on the titles of manuscripts published in the Special Issue, “Advances in Population-Based Healthcare Research: From Measures to Evidence”.

In this frame, the presented Special Issue of the *International Journal of Environmental Research and Public Health* offers insights into fundamental research on population health. A significant collection of contributions and studies is presented. Each of them adds interesting findings to meet the evolving challenges of population health, and suggests future directions for further research in the field. The collected articles cover, indeed, a wide range of thought-provoking topics (Figure 1): overall, 15 manuscripts were submitted to this Special Issue to be considered for publication. Of these, 10 papers were accepted for publication after evaluation by the Guest Editors and by many experts involved in the peer-review process.

Here, we analyse all the contributions according to their order of publication.

The first Special Issue paper explored the burden of cancer mortality attributable to carcinogenic infections in Italy, which accounted for 8.3% of total cancer deaths. Four carcinogenic infectious agents—*Helicobacter pylori*, hepatitis B virus (HBV), hepatitis C virus (HCV), and high-risk human papillomavirus (HPV) types—were found to be responsible for the vast majority of this burden. Since one-twelfth of cancer deaths were attributable to risk factors which are both preventable and treatable, this research provided data that may inform policies and promote targeted interventions to reduce the impact of infections on cancer mortality [3].

Hajek and König identified an unexplored association between post-materialism—the transformation of individual values that emphasize self-expression and quality of life over economic and physical security—and the frequency and reason for doctor visits. Compared with materialism, authors found a reduction in doctor visits associated with post-materialism, both in the total sample and women. Although the male subsample was rather small to reach firm conclusions, in post-materialistic women, visiting doctors was less likely for chronic illnesses and more likely for reasons of preventive medical check-up/vaccination. The study offers thought-provoking insights for further research to confirm results and understand underline mechanisms [4].

In their research, Khodabandeh et al. developed a detailed mathematical model aimed at remodelling traditional home healthcare organization with the goal of minimizing total costs. By considering a travel balancing hypothesis, the authors proposed a straight-forward model that could be exploited to minimize the downgrading costs, which characterize the difference between the potential and actual skills of the nurses. The study also provides useful insights that are helpful to inform managerial decisions to better allocate available resources [5].

Readmission after discharge from long-term home care leads to a relevant health burden and considerable costs. Su et al. suggested the application of the LACE index for readmission, a tool normally used to predict 30-day readmission or death in patients in hospital wards. Although accuracy issues need to be better addressed through further analysis, the strategy seems to halve readmission of long-term home care patients, with the highest effect for infection-related problems [6].

Keeves et al. analysed allied health professionals’ (AHPs) awareness and perceptions towards factors impacting the access to healthcare for people following serious injury in Australia. Participants confirmed that barriers existed in both urban and regional areas, being particularly prevalent in the latter. Major barriers were related to the complexity of funding systems and health services. Authors suggested that more research should explore which improvements could ensure equitable access to healthcare, while also considering the heterogeneity of the trauma population [7].

Kim et al. conducted a nested case–control study using Korean national health screening data to investigate the association between anaemia and an increased risk of osteoporosis, two globally widespread conditions in women [8,9]. The findings suggested a correlation between low haemoglobin levels and increased prevalence of osteoporosis, marking the importance of correcting low haemoglobin to prevent osteoporosis, especially in the population with comorbidities [8].

In an another study, the same authors also investigated the association between statin use and the occurrence of Meniere’s disease, considering previous evidence of statins having a protective effect against inner ear diseases. The findings did not support this relationship in the adult population, though a possible inverse association of previous lipophilic statin use with Meniere’s disease was suggested [10].

Around 1.3 billion people use tobacco worldwide, and more than 7 million die prematurely every year due to smoking [11]. Despite the massive health burden of tobacco-related diseases, many substance use treatment centres do not offer tobacco use interventions (i.e., screening and treatment): in this sense, Le et al. documented changes in the provision of each of the five As of tobacco intervention—namely, asking about tobacco use; advising to quit; assessing willingness to quit; assisting with quitting; and arranging follow-up—before and after the implementation of a comprehensive tobacco-free workplace program amongst clinicians from substance use treatment centres in Texas, USA. The authors highlighted the importance of organizational factors for promoting clinician behaviour changes to tackle the tobacco epidemic [12].

To learn how socio-environmental characteristics influence mental health outcomes differently among women and men [13], Gupta et al. documented individual- and neighbourhood-level sex differences in mental health service use in a context of uniquely smaller urban and rural settlements. The results observed sex-specific neighbourhood deprivation surrounding women’s risk of mental health service use compared with men, calling for specific community interventions to reduce inequalities in mental service utilization and, more in general, in mental health burdens [14].

Lastly, Bae’s research aimed to identify factors that affect absenteeism and presenteeism in the Korean working population, suggesting that workers’ quality of life and productivity could be improved by focusing on factors including fatigue, high temperatures at the workplace, and lower back pain as predictors of presenteeism [15].

## 2. Conclusions

Despite the variety and heterogeneity of the topics discussed in the presented articles, this Special Issue contributes both to advancing and stimulating the scientific debate about population health, and also enriches the current body of evidence, implications, and impacts. Taken together, all contributions emphasize that research needs to better integrate health outcomes’ distribution to inform health interventions and policies.
